# Combined absence of TRP53 target genes ZMAT3, PUMA and p21 cause a high incidence of cancer in mice

**DOI:** 10.1038/s41418-023-01250-w

**Published:** 2023-12-18

**Authors:** Margs S. Brennan, Kerstin Brinkmann, Gerard Romero Sola, Geraldine Healey, Leonie Gibson, Lahiru Gangoda, Margaret A. Potts, Elizabeth Lieschke, Stephen Wilcox, Andreas Strasser, Marco J. Herold, Ana Janic

**Affiliations:** 1 The Walter and Eliza Hall Institute of Medical Research (WEHI), 1G Royal Parade, Parkville, Melbourne, VIC 3052 Australia; 2 Department of Medical Biology, The University of Melbourne, Melbourne, VIC Australia; 3 Department of Medicine Huddinge, Centre for Haematology and Regenerative Medicine, Karolinska Institutet, Stockholm, Sweden; 4 Department of Medicine and Life Sciences, Universidad Pompeu Fabra, Barcelona, Spain; 5 Genome Engineering and Cancer Modelling Program, Olivia Newton-John Cancer Research Institute, Melbourne, VIC Australia; 6 School of Cancer Medicine, La Trobe University, Melbourne, VIC Australia

**Keywords:** Cancer models, Cancer genetics, Cancer

## Abstract

Transcriptional activation of target genes is essential for TP53-mediated tumour suppression, though the roles of the diverse TP53-activated target genes in tumour suppression remains poorly understood. Knockdown of ZMAT3, an RNA-binding zinc-finger protein involved in regulating alternative splicing, in haematopoietic cells by shRNA caused leukaemia only with the concomitant absence of the PUMA and p21, the critical effectors of TRP53-mediated apoptosis and cell cycle arrest respectively. We were interested to further investigate the role of ZMAT3 in tumour suppression beyond the haematopoietic system. Therefore, we generated *Zmat3* knockout and compound gene knockout mice, lacking *Zmat3* and *p21*, *Zmat3* and *Puma* or all three genes. *Puma*^*−/−*^*p21*^*−/−*^*Zmat3*^*−/−*^ triple knockout mice developed tumours at a significantly higher frequency compared to wild-type, *Puma*^*−/−*^*Zmat3*^*−/−*^ or *p21*^*−/−*^*Zmat3*^*−/−*^deficient mice. Interestingly, we observed that the triple knockout and *Puma*^*−/−*^*Zmat3*^*−/−*^ double deficient animals succumbed to lymphoma, while *p21*^*−/−*^*Zmat3*^*−/−*^ animals developed mainly solid cancers. This analysis suggests that in addition to ZMAT3 loss, additional TRP53-regulated processes must be disabled simultaneously for TRP53-mediated tumour suppression to fail. Our findings reveal that the absence of different TRP53 regulated tumour suppressive processes changes the tumour spectrum, indicating that different TRP53 tumour suppressive pathways are more critical in different tissues.

## Introduction

The tumour suppressor TP53 (mouse TRP53, often referred to as p53) is a frequently mutated gene in human cancer [1–3]. Although TP53-mediated transcriptional regulation of diverse cellular responses is known to be critical for its ability to prevent the development of cancer, the role of many TP53 target genes in tumour suppression remains unclear [2, 4]. Recent studies in mice sought to identify the mechanisms that are critical for TRP53-mediated tumour suppression, using sensitised shRNA gene knock-down and CRISPR/Cas9 knockout screens in cells in culture and even in vivo [5, 6]. Amongst the hits identified in these screens, the RNA binding protein ZMAT3 [7, 8] was found to be a potent tumour suppressor. ZMAT3 (also known as WIG-1) is an RNA-binding zinc-finger protein that is involved in regulating alternative splicing and as such is expressed in a broad range of tissues [6]. ZMAT3 was reported to control splicing of mRNAs, including those encoding the negative regulators of TRP53 function, MDM2 and MDM4, several other splicing factors (e.g., HNRNPDL, DHX9) and the cell adhesion and stem cell marker CD44 [6, 9]. ZMAT3 has been described to be under the direct transcriptional control of TRP53 in various cell types [5, 6, 8, 10], indicating its broad role for tumour suppression in diverse tissues. The absence of TRP53 causes a reduction in *Zmat3* expression in a range of human carcinomas (e.g., breast, lung) and high levels of *Zmat3* expression in malignant cells predict increased patient survival in certain cancers [6]. Moreover, an analysis of the Project Achilles CRISPR/Cas9 gene knockout screening data in human cancer cells revealed that ZMAT3 inactivation enhances proliferation of malignant cells with wild-type (wt) TP53. This further supports a role for ZMAT3 in tumour suppression acting downstream of TP53 [6]. Of note, CRISPR/Cas9-mediated loss of ZMAT3 enhanced tumorigenesis in autochthonous mouse models of lung adenocarcinoma and hepatocellular carcinoma [6]. However, unlike loss of TRP53, the absence of ZMAT3 did not have marked impact on the rate of tumour development or severity of malignant disease in the context of murine c-MYC*-*driven lymphomagenesis or mutant *Kras*^*G12D*^-driven lung adenocarcinoma development [11]. This indicates that the relative importance of ZMAT3 in TRP53-mediated tumour suppression may vary depending on cell type and/or the context of other oncogenic drivers present in the emerging neoplastic cell population. In vivo studies have shown that shRNA-mediated silencing of ZMAT3 in haematopoietic stem/progenitor cells (HSPCs) caused development of leukaemia/lymphoma in transplant recipient mice only when PUMA and p21, the critical effectors of TRP53-mediated apoptosis [12, 13] and cell cycle arrest [14] respectively, were also absent [5]. A limitation of these studies was that ZMAT3, PUMA and p21 were removed only in the haematopoietic compartment. Therefore, it remains unclear what impact the absence of ZMAT3 either on its own, or in conjunction with the loss of TRP53 induced apoptosis and cell cycle arrest/cell senescence might have in other tissues. To address this question, we generated mice lacking ZMAT3, PUMA and p21. We found that these triple knockout (TKO) mice spontaneously developed malignancy at a considerably higher frequency compared to wt controls as well as *Puma*^*−/−*^*Zmat3*^*−/−*^ and *p21*^*−/−*^*Zmat3*^*−/−*^ double knockout (DKO) mice. Most of the tumours from the TKO mice were of haematopoietic cell origin. These findings demonstrate that this combination of defects in TRP53-activated cellular responses, even if present in all cells, predominantly causes leukaemia/lymphoma. They also reaffirm that defects in multiple TRP53-activated cellular responses must collude to drive tumorigenesis.

## Results

### Adult pre-neoplastic *Puma*^*−/−*^*p21*^*−/−*^*Zmat3*^*−/−*^ mice have only minor abnormalities in their haematopoietic cell populations

**Fig. 1:**
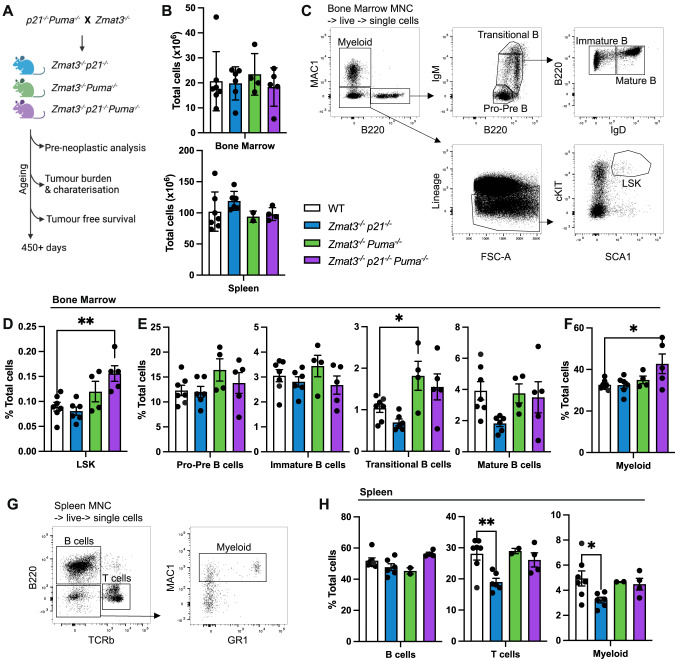
Lymphoid organ analysis of young adult mice of the indicated genotypes. **A** Schematic for tumour development study. Mouse cohorts were analysed for pre-neoplastic phenotypes and monitored for tumour-free survival. **B**–**H** Single-cell suspensions were prepared from spleen, thymus, peripheral blood and bone marrow of *Puma*^*−/−*^*p21*^*−/−*^*Zmat3*^*−/−*^ (*N* = 5), *Puma*^*−/−*^*Zmat3*^*−/−*^ (*N* = 2–4), *p21*^*−/−*^*Zmat3*^*−/−*^ (*N* = 6), and wt (*N* = 7) mice and the indicated haematopoietic cell subsets were examined by immunostaining and FACS analysis. **B** Total cell counts for bone marrow (1 femur, top) and whole spleen (bottom). **C** Representative FACS plots from wt mice that indicate gating strategy to identify the cell populations of interest in the bone marrow: myeloid (MAC1^+^), LSK (Lineage^-^SCA1^+^cKIT^+^) haematopoietic stem/progenitor cells (HSPCs) and B lymphoid cells; pro-pre (B220^lo^IgM^-^), immature (B220^+^IgM^lo^ IgD^-^), transitional (B220^+^IgM^hi^) and mature (B220^+^IgM^med^IgD^+^) B lymphoid cells. The lineage marker antibody cocktail included antibodies against NK1.1, TER119, Ly6G, F4/80, CD2, CD4 and CD8. **D**–**F** Percentages of the indicated cell subsets in the bone marrow from mice of the indicated genotypes. **G** Representative FACS plots of wt mice indicate gating strategy to identify cell populations in the spleen: B cells (B220^+^), T cells (TCRβ^+^) and myeloid cells (MAC1^+^B220^-^TCRβ^-^). **H** Percentages of the indicated cell subsets in the spleen of mice of the indicated genotypes. Data represent mean ± SEM. Statistical significance was calculated by one-way ANOVA *p < 0.05. N number of mice, MNC mono-nuclear cells as determined by forward/side scatter. For data of analysis of single knockout control mice, *Puma*^*−/−*^ (*N* = 7)*, p21*^*−/−*^(*N* = 8)*, Zmat3*^*−/−*^ (*N* = 6), refer to Fig. S1.

To investigate the impact of combined loss of ZMAT3, PUMA and p21 in TRP53-mediated tumour suppression we crossed *Zmat3*^*−/−*^ with *Puma*^*−/−*^*p21*^*−/−*^ mice to obtain *Puma*^*−/−*^*Zmat3*^*−/−*^*, p21*^*−/−*^*Zmat3*^*−/−*^ and *Puma*^*−/−*^*p21*^*−/−*^*Zmat3*^*−/−*^ animals (Fig. 1A). *Puma*^*−/−*^*Zmat3*^*−/−*^, *p21*^*−/−*^*Zmat3*^*−/−*^ and *Puma*^*−/−*^*p21*^*−/−*^*Zmat3*^*−/−*^ offspring were born at the expected Mendelian ratios of inheritance observed for heterozygous crosses of each allele within the colony (Table S1), as well as from *Puma*^*+/-*^*Zmat3*^*+/-*^ and *p21*^*+/-*^*Zmat3*^*+/-*^ di-hybrid inter-crosses (Table S2). The DKO and TKO mutant mice reached adulthood without any notable defects.

Previous reports have demonstrated important individual and overlapping roles of ZMAT3, PUMA and p21 in lymphoid cells [5, 12, 14]. Therefore, we hypothesised that *Puma*^*−/−*^*p21*^*−/−*^*Zmat3*^*−/−*^ TKO mice may display a marked pre-leukaemic phenotype with a pronounced expansion of certain haematopoietic cell populations. We therefore determined the overall composition of the haematopoietic system of the TKO, and both DKO mice by immunostaining and fluorescence-activated cell sorter (FACS) analysis at 8–12 weeks of age. The cellularity of the different lymphoid organs (e.g. spleen, bone marrow, thymus) in the DKO and TKO mice were comparable to those in wt controls (Figs. 1B, S1A), as were the white blood cell (WBC) counts in peripheral blood (PB) (Fig. S1B). T cell development in the thymus appeared normal with the overall distribution of the double negative (DN, CD4^-^CD8^-^ progenitors), double-positive (DP, CD4^+^CD8^+^ immature) and single positive CD4^+^ and CD8^+^ (mature) thymocytes comparable between the DKO as well as TKO mice with those seen in wt controls (Fig. S1C, D). In the bone marrow, we observed a small but significant increase in the immature stem and multi-potent progenitor population (termed LSK, defined by lack of any mature lineage markers and expression of both SCA1 and cKIT) in the *Puma*^*−/−*^*p21*^*−/−*^*Zmat3*^*−/−*^ TKO mice compared to wt controls (Fig. 1C, D). However, this did not result in any differences in B-cell development in the bone marrow, with only a small, yet significant increase in myeloid cells observed (Figs. 1E, F, S1E, F). The numbers of all mature cell types in the spleen that we tested were comparable between DKO as well as TKO mice with those seen in wt controls, with comparable frequencies of B cells (B220^+^), T cells (TCRβ^+^) and myeloid cells (MAC1^+^) (Figs. 1G, H, S1G–I). In addition, histological analysis of major organs revealed no lesions of significance in young adult DKO and TKO mice (Fig. S2). Overall, these findings demonstrate that the combined absence of ZMAT3, PUMA and p21 does not cause marked defects in pre-cancerous mice.

### Cells from *Puma*^*−/−*^*p21*^*−/−*^*Zmat3*^*−/−*^ mice are resistant to TRP53-mediated apoptosis triggered by DNA damage

**Fig. 2:**
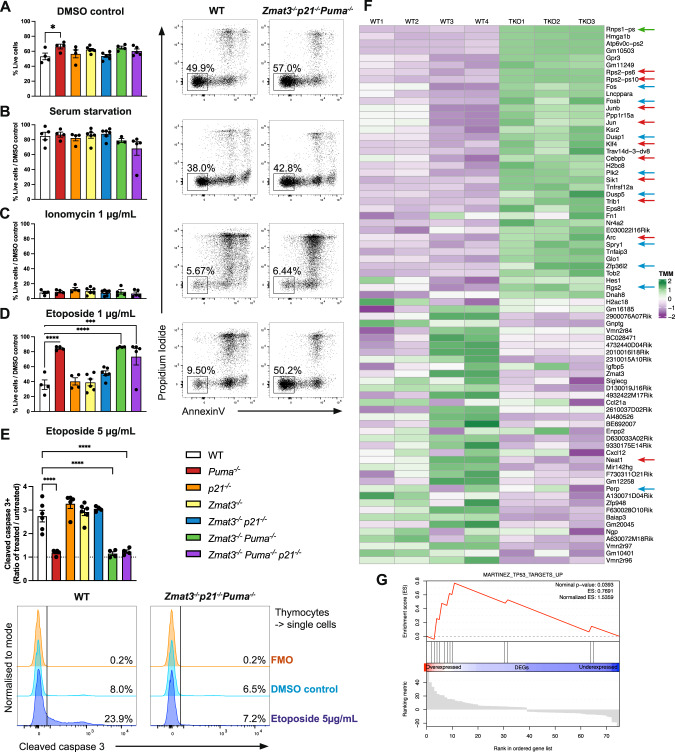
Impact of combined loss of ZMAT3, PUMA and p21 on thymocyte transcriptional landscape and survival upon treatment with various apoptotic stimuli. Thymocytes from mice of the indicated genotypes were isolated and either (**A**) left untreated (DMSO control), (**B**) treated with 1 µg/mL ionomycin, (**C**) exposed to serum deprivation in culture or (**D**) treated with 1 µg/mL etoposide. Cell viability was assessed after 24 h by Annexin-V/PI staining and FACS analysis. Data are presented as mean ± SEM of Annexin-V-PI- population (live cells) (**A**) and live cells relative to DMSO treated control samples (**B**–**D**). Representative FACS plots are shown to the right of each graph. **E** Thymocytes were treated with 5 µg/mL etoposide for 6 h, fixed, followed by intracellular staining for activated (i.e. cleaved) caspase-3 (CC3) and then subjected to FACS analysis. Data are presented as ratio of CC3+ cells in treated *vs* untreated (DMSO control) thymocytes with representative histograms shown below. FMO = fluorescence minus one staining control. Statistical significance was calculated by one-way ANOVA *p = 0.035, ***p = 0.0009, ****p < 0.0001. **F**, **G** RNAseq analysis of isolated thymocytes from 8–12 week old wt (*N* = 4) and *Puma*^*−/−*^*p21*^*−/−*^*Zmat3*^*−/−*^ TKO (*N* = 3) mice. **F** Heat map showing all significant differentially expressed (DE) genes between thymocytes from wt and TKO mice. Each column represents data from an individual mouse. Data are colour coded by gene expression Z-scores. Arrows indicate genes that regulate TRP53 and are reported to compromise its tumour suppression function (red), known TRP53 target genes (blue) and splicing regulators (green). **G** Gene-set enrichment analysis (GSEA) on DE genes between thymocytes from wt and TKO mice shows significant overlap with the Mouse Gene Set: MARTINEZ_TP53_TARGETS_UP [65].

The BH3-only protein PUMA is critical for apoptosis induced by stress stimuli that activate TRP53, such as DNA damage [12, 13, 15]. To verify that cells from TKO mice were, as expected, resistant to TRP53-mediated apoptosis, we exposed thymocytes from *Puma*^*−/−*^*p21*^*−/−*^*Zmat3*^*−/−*^ as well as *Puma*^*−/−*^*Zmat3*^*−/−*^*, p21*^*−/−*^*Zmat3*^*−/−*^, *Zmat3*^*−/−*^, *p21*^*−/−*^, *Puma*^*−/−*^ and wt mice to apoptosis inducing agents in vitro and their survival was then measured by flow cytometric analysis. The survival of *Puma*^*−/−*^*p21*^*−/−*^*Zmat3*^*−/−*^ TKO thymocytes was comparable to that of DKO and wt thymocytes when either left untreated (DMSO control), treated with ionomycin or starved of serum (growth factor deprivation) (Fig. 2A–C). As expected, thymocytes from TKO and *Puma*^*−/−*^*Zmat3*^*−/−*^ DKO mice were resistant to etoposide, a cytotoxic stimulus that kills these cells via a TRP53-dependent process. At 24 h, there was less than 40% survival of wt thymocytes but more than 80% survival of the TKO, *Puma*^*−/−*^*Zmat3*^*−/−*^ DKO and *Puma*^*−/−*^ thymocytes (Fig. 2D). To confirm that this survival advantage was due to failure to induce apoptosis owing to the absence of PUMA, we performed cleaved caspase-3 (CC3) staining, a marker for cells that have initiated apoptosis, after etoposide treatment, followed by flow cytometric analysis. At 24 h after treatment a drastic decrease in CC3 staining in *Puma*^*−/−*^*p21*^*−/−*^*Zmat3*^*−/−*^ as well as *Puma*^*−/−*^*Zmat3*^*−/−*^ and *Puma*^*−/−*^ thymocytes was observed compared to wt thymocytes (Fig. 2E). These data show that cells from TKO mice are resistant to TRP53-driven apoptosis triggered by DNA damage due to the absence of PUMA, but are normally sensitive to several TRP53-independent apoptotic stimuli.

### ZMAT3 impacts expression of genes involved in TRP53 mediated regulation of the haematopoietic compartment

Given the importance of ZMAT3 as an RNA binding protein we performed RNA-seq analysis on cells from young adult (8–12 weeks old) age-matched *Puma*^*−/−*^*p21*^*−/−*^*Zmat3*^*−/−*^, *Zmat3*^*−/−*^ and wt control mice. Comparison of gene expression profiles of *Puma*^*−/−*^*p21*^*−/−*^*Zmat3*^*−/−*^ and wt thymocytes revealed minor changes in the transcriptional landscape and identified 75 significantly differentially expressed (DE) genes (Figs. 2F, G, S3A, B, Table S3). GO term annotation uncovered various categories related to mechanisms that cells use to control gene expression and maintain cellular proliferation, differentiation, or cell adhesion (Fig. S3A). Notably, the most statistically DE genes in *Puma*^*−/−*^*p21*^*−/−*^*Zmat3*^*−/−*^ cells included genes that regulate TP53 and compromise its tumour suppression function (Fig. 2G, red arrows), such as Rps2 - regulator of TRP53-MDM2 signalling [16], *Klf4* [17], *Trb1* [18], *Neat1* [19], *Cebpb* [20], *Sik1* [21], *Jun* [22], *Arc* [23], and some known TRP53 target genes (Fig. 2G, blue arrows), including *Dusp1* [24], *Perp* [25], *Dusp5* [26], *Fos* [27], *Rgs2* [28], *Spry1* [29], *Plk2* [30] and *Zfp36L2* [31]. Additionally, gene-set enrichment analysis (GSEA) comparing wt with *Puma*^*−/−*^*p21*^*−/−*^*Zmat3*^*−/−*^ thymocytes showed a significant enrichment for TRP53 regulated genes (Fig. 2G). Thus, these data indicate that ZMAT3 may function in tumour suppression by regulating TRP53 function. To assess the contribution of PUMA and p21 to the observed transcriptomic changes, we compared gene expression profiles between *Puma*^*−/−*^*p21*^*−/−*^*Zmat3*^*−/−*^ and *Zmat3*^*−/−*^ thymocytes. This revealed only 9 significantly DE genes (Fig. S3C), with Rnps1 and Rps2 being the only genes overlapping between the two sets of comparisons (Fig. S3B, C). This demonstrates that the absence of PUMA and p21 have only minor impact on the transcriptome changes seen in *Puma*^*−/−*^*p21*^*−/−*^*Zmat3*^*−/−*^ cells. Collectively, our findings support the model in which ZMAT3 regulates the expression of a range of genes involved in TRP53 regulation of various cellular processes.

### Consequences of combined loss of ZMAT3 and p21 on γ-radiation induced thymic lymphoma development

**Fig. 3:**
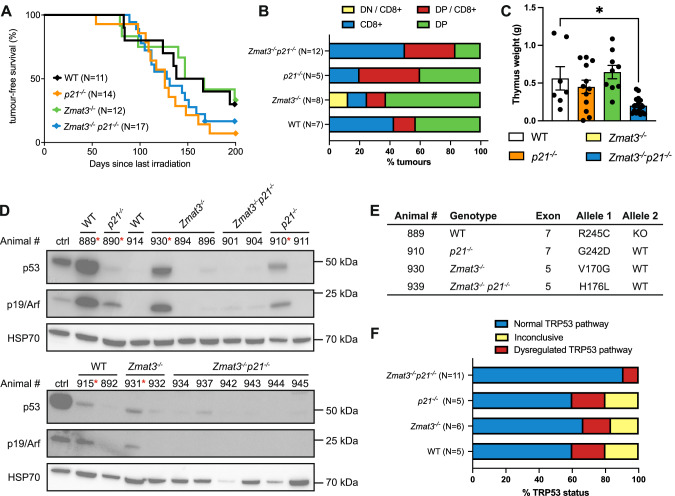
The combined absence of ZMAT3 and p21 does not accelerate γ-radiation induced thymic lymphoma development. **A** Kaplan-Meier curves showing percentages of tumour-free mice of the indicated genotypes after exposure to four weekly doses of γ-radiation (1.5 Gy for each dose). Differences in thymic lymphoma incidence between wt and *p21*^*−/−*^*Zmat3*^*−/−*^ were not statistically significant. *P* value determined by log-rank (Mantel-Cox) test *p* = 0.5. **B** Immunophenotyping of γ-radiation-induced thymic lymphomas arising in mice of the indicated genotypes, as assessed by cell surface marker staining and flow cytometric analysis of tumour cells from the thymus. Data are presented as the frequency of the indicated phenotypes for each genotype. Double negative CD4^-^CD8^-^ (DN) or double positive CD4^+^CD8^+^ (DP) lymphoma cells found in the thymus that show a trend towards the CD8 T cell lineage (DP/CD8^+^ or DN CD8^+^, respectively). N number of mice. **C** Thymus weights from sick mice of the indicated genotypes. Significant differences were observed in spleen weights between the sick *p21*^*−/−*^*Zmat3*^*−/−*^ and sick wt γ-irradiated mice. *p21*^*−/−*^*Zmat3*^*−/−*^ (*N* = 12), *p21*^*−/−*^ (*N* = 7)*, Zmat3*^*−/−*^ (*N* = 7) and wt (*N* = 5). Mean ± SEM, Unpaired Students *t*-test **p = 0.0065. **D** Western blot analysis of p19ARF, TRP53 and HSP70 (loading control) in thymic lymphomas of the indicated genotypes. Red asterisk indicates samples with dysregulated TRP53 pathway. The *Trp53* mutant *Eµ-Myc* lymphoma cell line EMRK1172 [51] was used as a control for p19ARF and mutant TRP53 protein overexpression. Protein size standards in kilodaltons (kDa) are indicated. The numbers represent the identification of individual mice in the colony. **E** *Trp53* exons 4-11 were sequenced from tumours (*N* = 26, Table S4). Table summary of mutations found, listed by amino acid change, *Trp53* knock out (KO) or *Trp53* wild-type (WT) alleles. **F** Summary of the TRP53 status in γ-radiation-induced thymic lymphomas arising in mice of the indicated genotypes as assessed by Western blot analysis and/or *Trp53* exon sequencing (see also Table S4). Inconclusive refers to lymphomas where TRP53 pathway dysregulation was seen by only Western blot analysis or exon sequencing but not in both assays.

Exposure of mice from a young age (starting at ~4 weeks) to repeated low-dose γ-radiation drives thymic lymphoma development through a mechanism that is suppressed by TRP53 [32, 33]. To investigate whether TRP53-mediated induction of ZMAT3 could restrain γ-radiation induced thymic lymphoma development, we examined the consequences of individual or combined loss of ZMAT3 and p21 in this model. Mice lacking PUMA alone or in combination with ZMAT3 and p21 were not included as it has been previously shown that mice defective in TRP53-induced apoptosis due to loss of PUMA are profoundly resistant to γ-radiation-induced thymic lymphoma development [33, 34]. We found that *p21*^*−/−*^*Zmat3*^*−/−*^ as well as *Zmat3*^*−/−*^ and *p21*^*−/−*^ mice developed thymic lymphoma at a similar rate to wt mice (Fig. 3A). Immunophenotyping of the tumours from sick *p21*^*−/−*^*Zmat3*^*−/−*^, *Zmat3*^*−/−*^ and *p21*^*−/−*^ mice confirmed that these lymphomas were all of T lymphoid origin expressing THY1 and variable expression of CD4 and CD8 (Figs. 3B, S4A). Lymphoma burden, as determined by thymus weight, was smaller in *p21*^*−/−*^*Zmat3*^*−/−*^ mice compared to those detected in *Zmat3*^*−/−*^, *p21*^*−/−*^ and wt mice (Fig. 3C). However, no differences between these genotypes were seen in spleen weights or white blood cell counts (Fig. S4B, C), consistent with no difference in tumour burden. Interestingly, high levels of p19ARF which are indicative of loss of TRP53 pathway function, were apparent in 2 out of 4 wt, 2 out of 5 *Zmat3*^*−/−*^, 2 out of 3 *p21*^*−/−*^ but none out of 8 *p21*^*−/−*^*Zmat3*^*−/−*^ lymphomas tested by Western blotting (Fig. 3D). We therefore sequenced *Trp53* exons in all available tumours and found only 1/11 *p21*^*−/−*^*Zmat3*^*−/−*^ tumours to harbour a *Trp53* mutation, with considerable higher frequencies seen in lymphomas from the other tested genotypes, *p21*^*−/−*^ or *Zmat3*^*−/−*^ (Fig. 3E, F, Table S4). These findings demonstrate that in this mouse model of lymphomagenesis tumour suppression by TRP53 does not depend on ZMAT3 and/or p21 function. However, the reduction of readily detectable defects in TRP53 pathway function in the lymphomas from the *p21*^*−/−*^*Zmat3*^*−/−*^ mice indicates that the absence of these two TRP53 target genes may obviate the selection for mutations in *Trp53* in this malignant disease.

### *Zmat3*^*−/−*^*Puma*^*−/−*^*p21*^*−/−*^ mice are prone to spontaneous tumour development

**Fig. 4:**
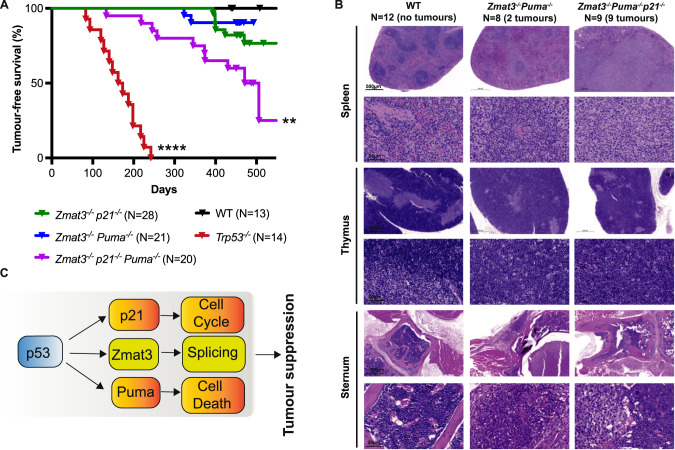
The combined absence of ZMAT3, PUMA and p21 causes spontaneous tumour development in mice. **A** Kaplan-Meier curves showing tumour-free survival of *Puma*^*−/−*^*p21*^*−/−*^*Zmat3*^*−/−*^, *Puma*^*−/−*^*Zmat3*^*−/−*^ and *p21*^*−/−*^*Zmat3*^*−/−*^ mice. Data from wt and *Trp53*^*−/−*^ mice are included as controls. Log-rank (Mantel-Cox) test P values comparing to wt mice; *Puma*^*−/−*^*p21*^*−/−*^*Zmat3*^*−/−*^ *p* = 0.003**; *Puma*^*−/−*^*p21*^*−/−*^ *p* = 0.06; *Puma*^*−/−*^*Zmat3*^*−/−*^ *p* = 0.26; ****p < 0.0001. **B** Representative H&E-stained sections of spleen, bone marrow (sternum) and thymus of sick *Puma*^*−/−*^*p21*^*−/−*^*Zmat3*^*−/−*^ (#454,615) and *Puma*^*−/−*^*Zmat3*^*−/−*^ (#471,624) mice, and an age-matched healthy wt (#953) mouse for comparison. Scale bar denotes 500 µm or 20 µm, respectively. N number of mice of a particular genotype analysed. **C** Model: ZMAT3 loss drives spontaneous tumour development in collaboration with additional loss of PUMA and p21.

To examine the impact of combined loss of ZMAT3, PUMA and p21 in all tissues on the development of cancer, we aged *Puma*^*−/−*^*p21*^*−/−*^*Zmat3*^*−/−*^ TKO as well as *Puma*^*−/−*^*Zmat3*^*−/−*^, *p21*^*−/−*^*Zmat3*^*−/−*^ and as controls *Trp53*^*−/−*^ as well as wt mice for 450–500 days. These animals were monitored for tumour development and major tissues were collected either at the time of sickness or at 450–500 days (study endpoint) regardless of health status. Interestingly, TKO as well as *Puma*^*−/−*^*Zmat3*^*−/−*^ and *p21*^*−/−*^*Zmat3*^*−/−*^ DKO mice were significantly more prone to spontaneous tumour development compared to wt controls, with TKO mice showing a cancer incidence of nearly 50% by 500 days (Fig. 4A). The majority of sick *Puma*^*−/−*^*Zmat3*^*−/−*^ and *Puma*^*−/−*^*p21*^*−/−*^*Zmat3*^*−/−*^ mice presented with lymphoma (Figs. 4B, S5, Table 1) as determined by histopathological analysis, while most sick *Zmat3*^*−/−*^*p21*^*−/−*^ mice presented with solid tumours (Table 1). Consistent with previous reports [35], *Trp53*^*−/−*^ mice all develop cancer, mostly thymic lymphomas, within 250 days of age [35] (Fig. 4A, Table 1). None of the control wt mice developed tumours during the 500 day observation period (Fig. 4A, B). Collectively, these results show that the combined absence of the three TRP53 target genes *Zmat3*, *Puma* and *p21* causes a high incidence of spontaneous tumour development. It also shows that most malignancies arising in the compound mutant mice lacking PUMA are lymphomas whereas mice lacking ZMAT3 and p21 develop solid cancers.

**Table 1:** Tumour types observed in *Zmat3*, *Puma*, *p21* compound mutant mice.

Animal #	Sex	Age (days)	Tumour type(s) observed
*Zmat3*^*−/−*^*;Puma*^*−/−*^			
624	M	340	Early B- (Spl) and T-cell (Thy) lymphoma, infiltration (Liv, Kid, SC)
471	M	323	B-cell (Spl) and T-cell (Thy) lymphoma, metastasis (Liv, Kid)
	6 M/F	450–493	No tumours observed
	13 M/F	450–469	N.D. No tumours observed
*Zmat3*^*−/−*^*;p21*^*−/−*^			
222	M	572	Hepatocellular carcinoma
276	M	399	Hepatocellular carcinoma
283	F	470	Sarcoma
285	F	423	Sarcoma
297	F	394	Sarcoma
293	M	400	Subcutaneous tumour resembling amelanotic melanoma
831	M	400	Lymphoma (Spl), metastasis (Liv, Kid)
832	M	549	N.D. Enlarged spleen (0.3 g), mottled liver (0.26 g) observed
	2 M/F	461–517	No tumours observed
	18 M/F	459–517	N.D. No tumours observed
*Zmat3*^*−/−*^*;p21*^*−/−*^*;Puma*^*−/−*^			
615	M	245	Early B-cell (Spl) and T-cell (Thy, MLN) lymphoma
616	M	257	Early B-cell (Spl) and T-cell (Thy) lymphoma
696	F	133	Early B-cell (Spl) and T-cell (Thy) lymphoma
454	F	430	B-cell lymphoma (Spl), myeloid tumour (SC/BM)
473	F	345	B-cell lymphoma (Liv)
507	M	374	B-cell (Spl) and T-cell (MLN) lymphoma, metastasis (L, Spl)
773	M	218	B-cell (Thy, LN) and T-cell (Spl) lymphoma
187	F	506	N.D. Splenomegaly (2.9 g) observed
651	F	453	Follicular hyperplasia (Spl)
562	F	373	Myelogenous leukaemia
	10 M/F	450–590	N.D, No tumours observed
WT			
	12 M/F	438–508	No tumours observed
*Trp53*^*−/−*^			
	14 M/F	83–424	N.D. 5x thymic lymphoma, 8x lymphoma, 1x solid tumour

Aged mice from *Zmat3*^*−/−*^*;p21*^*−/−*^ *Puma*^*−/−*^ inter-crosses were euthanised at ethical endpoint (sick, hind leg paralysis, tumour volume, anaemia). Wild-type (WT) and *Trp53*^−/−^ mice housed in the same facility are included as comparative controls. Tumour types were determined by pathological analysis of H&E-stained organs and tumour sections. Organs involved noted in brackets.

*Spl* spleen, *Thy* thymus, *Liv* liver, *Kid* kidney, *SC* spinal cord, *BM* bone marrow, *MLN* mesenteric lymph node, *L* lung, *LN* lymph node, *N.D*. histology not done, noted observations made at autopsy.

## Discussion

TP53 is a critical suppressor of cancer development and progression [1–3, 36]. TP53 functions as a transcription factor that can directly regulate the expression of ~500 target genes, and indirectly many more [4, 36, 37]. Direct transcriptional targets of TP53/TRP53 that are critical for the induction of apoptotic cell death, cell cycle arrest and senescence have been considered as essential effectors of TP53/TRP53-mediated tumour suppression [4]. Unexpectedly, however, genetic inactivation of these three TRP53-activated cellular processes, for example due to the combined absence of PUMA, NOXA and p21, does not render mice tumour prone [10, 38, 39]. In contrast, 100% of TRP53-deficient mice spontaneously develop tumours, on a C57BL/6 background mostly thymic T cell lymphoma, before 270 days of age [40, 41]. Genetic screens and genetically modified mouse strains have provided useful tools to examine the importance and biological functions of a range of TRP53 target genes in TRP53-mediated tumour suppression. Utilising in vivo CRISPR and shRNA screening in oncogene expressing mouse embryonic fibroblast in vitro [6] and haematopoietic stem/progenitor cells in vivo [5], respectively, identified the zinc-finger RNA-binding protein ZMAT3 as a critical factor contributing to TRP53-mediated tumour suppression.

Here we show that the combined absence of ZMAT3, PUMA and p21, the latter two critical for TRP53-mediated induction of apoptosis [12, 13] and cell cycle arrest/cell senescence [14], respectively, resulted in nearly 50% of spontaneous tumour development by 500 days of age. Given that PUMA is a key factor in TRP53-mediated apoptosis in haematopoietic cells [1, 12, 13, 15], it is perhaps not surprising that the majority of sick *Puma*^*−/−*^*Zmat3*^*−/−*^ and *Puma*^*−/−*^*p21*^*−/−*^*Zmat3*^*−/−*^ mice presented with lymphoma. Interestingly, some *Zmat3*^*−/−*^*p21*^*−/−*^ sick mice developed a range of solid cancers at a relatively old age. Their incidence of such malignant disease was significantly higher compared to wt mice. This indicates that loss of apoptotic function, cell cycle arrest/cell senescence and ZMAT3-regulated alternative splicing or not yet identified functions of ZMAT3 contribute to the tumour suppressive function of TRP53 in both haematological and solid cancers, with the combination of the genes lost determining cancer type. Notably, *Puma*^*−/−*^*Zmat3*^*−/−*^ and *Puma*^*−/−*^*p21*^*−/−*^*Zmat3*^*−/−*^ mice presented with lymphoma, and *Zmat3*^*−/−*^*p21*^*-/*^ mice mainly developed solid tumours (6/8). This indicates that the loss of PUMA is particularly critical for spontaneous development of haematopoietic malignancies, consistent with the profound impact of its absence on apoptotic death of lymphoid and myeloid cells [12, 13, 15].

Examination of the lymphoid tissues from young *Puma*^*−/−*^*Zmat3*^*−/−*^, *Zmat3*^*−/−*^*p21*^*−/−*^*Puma*^*−/−*^and *p21*^*−/−*^*Zmat3*^*−/−*^ mice revealed that they were largely normal, although we did observe a significant, albeit minor, increase in LSK HSPCs in *Puma*^*−/−*^*p21*^*−/−*^*Zmat3*^*−/−*^ mice compared to wt controls. This is consistent with earlier reports that the absence of TRP53 itself does not cause detectable abnormalities in the haematopoietic system of young healthy mice although it predisposes them to spontaneous lymphoma development [15, 40–42].

Several direct TRP53 target genes have been implicated in DNA damage-induced apoptosis signalling, but only the BH3-only proteins, PUMA and to lesser extent NOXA, have been shown to be essential for TRP53-induced apoptosis [12, 13, 15, 43]. As expected, thymocytes from *Puma*^*−/−*^*p21*^*−/−*^*Zmat3*^*−/−*^ and *Puma*^*−/−*^*Zmat3*^*−/−*^ mice displayed significant resistance to etoposide (comparable to thymocytes from *Puma*^*−/−*^ mice) to etoposide, a cytotoxic agent that kills these cells via a TRP53-dependent pathway [42, 44, 45]. This indicates that the additional loss of ZMAT3, alone or with further removal of p21, does not increase the protection against TRP53-dependent or TRP53-independent apoptosis that is caused by the absence of PUMA. Consistent with previous studies [5], this demonstrates that ZMAT3 does not have a prominent role in apoptosis, at least in lymphoid cells.

ZMAT3 is a double-stranded RNA binding protein and known TP53/TRP53 target gene, that has been shown to modulate splicing of genes involved in TP53/TRP53 regulation, various cellular processes, and splicing itself [46]. Similarly, we found here that ZMAT3 regulates expression of transcripts encoding proteins involved in the regulation of TP53/TRP53 function. The targets identified include, RPS2 is a ribosomal protein that was reported to interact with MDM2 to control the levels of TP53/TRP53 [16], SIK1 serine/threonine kinase that regulates TP53 function in apoptosis triggered by lack of adhesion to suppress breast cancer metastasis [21]. Additional genes included JUN, transcription factor and apoptosis repressor with caspase recruitment domain (ARC) that are commonly induced in cancers [22, 23]. Notably, CEBPB and KLF4 transcription factors were described to suppress the expression of TP53/TRP53 [20, 47] and frequently dysregulated in acute myeloid leukaemias [17]. In addition, we found that proteins encoded by known TP53/TRP53 target genes, including dual-specific threonine and tyrosine phosphatases DUSP1 [24] and DUSP5 [26], the FOS oncoprotein [27], RGS2 [28], SPRY1 [29], PLK2 [30] and ZFP36L2 [31] are regulated by ZMAT3. Collectively, these data indicate that ZMAT3 may suppress tumorigenesis by modulating the expression of the genes that control and maintain TP53/TRP53 activity.

Previous reports have shown that thymic T cell lymphoma development in mice induced by γ-radiation can be significantly accelerated by loss of *Trp53* (even loss of one allele of *Trp53*) [32, 34, 48]. This is likely due to impairment of DNA damage-induced apoptosis, cell cycle arrest/cell senescence and coordination of DNA repair, which constitute critical processes for TRP53-mediated tumour suppression [1–3, 36, 49, 50]. Whole body γ-irradiation activates TRP53 and causes induction of its downstream target genes *p21*, *Puma* and *Zmat3* in thymocytes as well as fibroblasts [5, 11] and probably many (if not all) other cell types. However, our investigations show that the absence of ZMAT3, either alone or in combination with loss of p21, did not accelerate (or slow) γ-radiation induced thymic T cell lymphoma development, in contrast to the loss of even a single allele of *Trp53*. Of note, while many *Zmat3*^*−/−*^ and *p21*^*−/−*^ thymic T cell lymphomas had acquired defects in TRP53 function, as revealed by high levels of p19ARF and DNA sequencing analysis, most *p21*^*−/−*^*Zmat3*^*−/−*^ lymphomas tested had retained wt TRP53 function. These findings demonstrate that ZMAT3 loss is not essential for the suppression of γ-radiation induced thymic T cell lymphoma development, probably because TRP53 can activate many at least partially redundant tumour suppressive processes in response to DNA damage.

In conclusion, our results confirm and extend the notion that ZMAT3 is a direct TRP53 target gene that contributes to TRP53-mediated tumour suppression. It probably does this by regulating a large number of mRNAs that function in the TP53/TRP53 network, thereby impacting, perhaps in a feed-forward activation loop, several TP53/TRP53-activated cellular responses [8, 46]. Of note, on its own loss of ZMAT3 does not cause cancer in mice but its tumour suppressive function becomes apparent when TRP53-mediated apoptosis (loss of PUMA) and/or cell cycle arrest/cell senescence (loss of p21) are concomitantly disabled. Similarly, the impact of loss of ZMAT3 in mouse models of lung and liver cancer was less pronounced than that caused by the absence of TRP53 [6, 11]. Additional support for the idea that in addition to the absence of ZMAT3, additional TP53/TRP53-regulated processes must also be disabled for spontaneous tumour development comes from the observation that mutations in *ZMAT3* are not prevalent in human cancers compared to the very high frequency of *TP53* mutations [6, 46]. Since the combined loss of ZMAT3, PUMA and p21 does not result in the same tumour spectrum and duration of tumour-free survival as loss of TRP53 alone. Hence other TP53/TRP53 regulated processes for tumour prevention such as coordination of DNA repair to maintain genome stability [5] are required. Thus, our findings further cement the importance of the coordinated action of multiple TP53/TRP53-activated cellular responses for tumour suppression. This may have implications for developing novel strategies for treating cancer as simultaneous activation of several of these responses might be needed for effective therapy.

## Materials and methods

### Mice

*Zmat3*^*−/−*5^, *Puma*^*−/−*^*p21*^*−/−*21^ and *Trp53*^*−/−*41^mice have been previously described. All mice were maintained on a C57BL/6-WEHI background. To produce *Puma*^*−/−*^*p21*^*−/−*^*Zmat3*^*−/−*^, *Puma*^*−/−*^*Zmat3*^*−/−*^ and *p21*^*−/−*^*Zmat3*^*−/−*^ mice, we serially inter-crossed *Zmat3*^*−/−*^ and *Puma*^*−/−*^*p21*^*−/−*^ mice. Genotyping was performed by polymerase chain reaction (PCR) to confirm the absence of *Zmat3, Puma* and/or *p21* genes (for PCR primers used, see Supplementary Table 5).

### Histology

Organs from mice were fixed in 10% buffered formalin solution and subsequently embedded in paraffin. Slides were prepared and stained with haematoxylin and eosin (H&E). Histological examination of the organs was performed by Phenomics Australia Histopathology and Slide Scanning Service, University of Melbourne.

### Thymic T cell lymphoma induction

*p21*^*−/−*^*Zmat3*^*−/−*^, *p21*^*−/−*^*, Zmat3*^*−/−*^ and wt mice (starting at 27 ± 6 days of age) were γ-irradiated weekly for 4 weeks with 1.5 Gy from a 60 Co source (Theratron Phoenix, Theratronics). Treated mice were then monitored for 200 days for signs of illness, and tumour onset was calculated from the last (4th) dose of γ-irradiation.

### *Trp53* exon sequencing

Genomic DNA was isolated from 5 × 10^6^ thymic lymphoma cells using the DNeasy Blood & Tissue Kit (Qiagen). A total of 9 PCR primers pairs spanning exons 4–11 of *Trp53* [51] were used to amplify coding regions. Subsequently, PCR products were subjected to a second PCR using indexing primers, purified using Ampure XP beads (Beckman Coulter), then pooled and sequenced using the MiSeq platform (Illumina).

### Immunostaining and flow cytometric analysis

Thymus, spleen, and bone marrow were harvested, and single cell suspensions were prepared in PBS (Gibco) with 5 mM EDTA (Merck), supplemented with 5% foetal calf serum (FCS; Sigma-Aldrich) for staining. Fluorochrome-conjugated monoclonal antibodies used are as follows; B220 (RA3-6B2), IgM (RMM-1), IgD (11.26C), TCRβ (H57.597), CD4 (GK1.5), CD8 (53-6.7), MAC1 (M1/70) SCA1 (E13-161.7), c-KIT (2B8), NK1.1(PK136), Ter119 (TER119), Ly6G (1A8-4-10-9), F4/80 (BM8), CD2 (RM2.1), and were obtained from eBioscience, BioLegend, or generated in-house. Staining with propidium iodide (PI Sigma-Aldrich; 1 μg/mL) was used to exclude dead cells. Whole-organ cell counts were determined by mixing a known concentration of APC Calibrite beads (Becton Dickinson) with each sample. Data were collected using LSRFortessa X-20 or FACSymphony analysers and examined using FlowJo 10 (Becton Dickson).

### Cell viability and activated (cleaved) caspase-3 assays

Thymi were harvested from mice of the indicated genotypes and single cell suspensions were prepared by gentle mashing through a 100 µm nylon strainer (Falcon). Cells were cultured in high-glucose Dulbecco’s modified Eagle’s medium (DMEM) supplemented with 10% foetal calf serum, 50 μM 2-mercaptoethanol, 100 mM asparagine, 100 U/mL penicillin, and 100 mg/mL streptomycin. For viability assay, 5 × 10^4^ cells were plated into 96-well flat-bottom plates in triplicate and treated, as described [35], with DMSO (control), 1 nM dexamethasone (Sigma-Aldrich), 1 µg/mL etoposide (Sigma-Aldrich), 1 µg/mL ionomycin (Sigma-Aldrich) or subjected to serum starvation by culturing in medium containing only 1% foetal calf serum. Cell viability was assessed at 24 h by resuspending cells in Annexin-V binding buffer (0.1 M Hepes (pH 7.4)), 1.4 M NaCl, 25 mM CaCl_2_ containing fluorescein isothiocyanate (FITC)-conjugated Annexin-V and 1 µg/mL PI. For activated (i.e. cleaved) caspase-3 staining, 5 × 10^4^ cells were plated into 96-well flat-bottom plates in triplicate and treated with 5 µg/mL etoposide for 6 h, fixed (FIX & PERM, Invitrogen), and stained with an antibody to detect cleaved caspase-3 conjugated to FITC (#9661, Cell Signalling). Cells were examined by flow cytometric analysis using a BD-Biosciences LSR-II analyser. Ten thousand events were recorded per sample and data were analysed using FlowJo 10 analysis software.

### Western blot analysis

For Western blot analysis, cell lysates were prepared in radio-immunoprecipitation assay (RIPA) buffer supplemented with PhosSTOP and complete protease inhibitor cocktail (Roche). Protein concentration was determined by Bradford assay using the Protein Assay Dye Reagent Concentrate (Bio-Rad, Hercules, CA). Samples of 10 μg of protein were prepared in Laemmli buffer, boiled for 5 min and size-fractionated by gel electrophoresis on NuPAGE 10% Bis-Tris 1.5-mm gels (Life Technologies) in 2-(N-morpholino) ethanesulfonic acid (MES) buffer. Proteins were then transferred onto nitrocellulose membranes (Life Technologies) using the iBlot membrane transfer system (Bio-Rad). Antibody dilution and blocking were performed in 5% skim milk, 0.1% Tween 20 in phosphate-buffered saline (PBS). The following antibodies were used for probing; mouse TRP53 (clone CM5, Novocastra), p19/ARF (clone 5.C3.1, Rockland) and HSP70 (clone N6, gift from Dr. R Anderson, Olivia Newton-John Cancer Research Institute, Melbourne, VIC, Australia), the latter used as a control for protein loading. Secondary antibodies used include goat anti-mouse IgG and goat anti rabbit-IgG, both conjugated to horseradish peroxidase (HRP) (Southern Biotech). Forte Western HRP substrate (Millipore, Billerica, MA) was used for developing the signal, and membranes were imaged and analysed using the ChemiDoc XRS1 machine with ImageLab software (Bio-Rad).

### RNA sequencing

RNA was extracted from thymocytes using the miRNeasy Micro Kit (Qiagen) according to the manufacturer’s protocol, including a DNase digestion step. An input of 100 ng total RNA for each sample was indexed separately using the TruSeq RNA Prep Kit v2 (Illumina). Each library was quantified using the Agilent Tapestation. The indexed libraries were pooled and diluted to 750 pM for paired end sequencing (2 × 116 cycles) on the NextSeq 2000 instrument using the P3 200 cycle High Output Kit (Illumina) according to the manufacturer’s instructions. The base calling and quality scoring were determined using Real-Time Analysis on board software v2.4.6, while the FASTQ file generation and de-multiplexing utilised bcl2fastq conversion software v2.15.0.4. Raw FASTQ files were processed, and quality control performed using the nf-core/rnaseq v3.10.1 [52, 53]. Reads were pseudo-aligned and quantified using the Salmon option [54]. The GRCm38.p6 version of the mouse reference genome and the GENCODE vM25 GTF annotation files were used. We filtered out genes: (i) with obsolete Entrez Gene IDs, (ii) ribosomal RNA (rRNA), (iii) non-protein coding immunoglobulin genes (iv) the *Xist* gene and (v) genes that are on chromosome Y. Next, we filtered out lowly expressed genes by discarding those that did not show a minimum reliable level of expression of 20 counts per million reads of the smallest library size, in at least all the samples of the smallest group [55, 56]. The DESeq2 package (v1.40.0) [55, 57] was used for the differential expression analysis. We adjusted for the sex as a batch effect correction and surrogate variables were calculated with SVA [58]. Genes with adjusted *p* < 0.05 (5% FDR) and absolute log2FC > 1.5 were considered as statistically significant. Functional enrichment analysis of the biological processes was conducted with the Gene Ontology (GO) database using the clusterProfiler package [59] GSEA [60] was carried out in R using the fgsea package v1.24.0 [61] using the hallmark and curated gene sets from the mouse MsigDb [60, 62–64] GSEA was used to test for enrichment of specific gene sets within a ranked list based on p-value and log2FC. Gene sets with a *p* < 0.05 were considered as statistically significant.

### Supplementary information

Supplemental Figures Legends

Supplemental Figure 1

Supplemental Figure 2

Supplemental Figure 3

Supplemental Figure 4

Supplemental Figure 5

Supplemental Table 1

Supplemental Table 3

Original Data File

PRE-Authorship form

## Data Availability

RNA-seq data have been deposited in the Gene Expression Omnibus (ncbi.nlm.nih.gov/geo) under accession number GSE248747. The code used for the RNA-Seq analysis is available at https://github.com/Geriroso/ZMAT3_analysis. All knockout mice will be made available to the scientific community upon request.
