# Supplementary material for: Combined absence of TRP53 target genes ZMAT3, PUMA and p21 cause a high incidence of cancer in mice
Source: Cell Death Differ. 2023 Dec 18;31(2):159–69. doi: 10.1038/s41418-023-01250-w (PMC10850490; doi:10.1038/s41418-023-01250-w)
Supplement: Supplementary file 1 — Supplemental Figures Legends [file 41418_2023_1250_MOESM1_ESM.docx]

**Supplementary Material**

**Supplementary Figure 1. Lymphoid organ analysis of young adult mice of the indicated genotypes.**

Single-cell suspensions were prepared from spleen, thymus, peripheral blood and bone marrow of *Puma^-/-^p21^-/-^Zmat3^-/-^* (N=5) , *Puma^-/-^Zmat3^-/-^* (N=2-4)*, p21^-/-^Zmat3^-/-^* (N=6), *Puma^-/-^* (N=7)*, p21^-/-^* (N=8)*, Zmat3^-/-^* (N=6) and wt (N=7) mice, and the indicated haematopoietic cell subsets were examined by immunostaining and FACS analysis. (**A**) Total cell counts for bone marrow (1 femur,) whole spleen and thymus in mice of the indicated genotypes. (**B**) Total white blood cell (WBC) counts in peripheral blood in mice of the indicated genotypes. (**C**) Representative FACS plot of cells from a wt mouse indicating the gating strategy to identify cell populations of interest in the thymus. Immature double-negative thymocytes (DN progenitor; CD4^-^CD8^-^), double-positive thymocytes (DP immature; CD4^+^CD8^+^) and the mature CD4^+^CD8^-^ and CD4^-^CD8^+^ single-positive T lymphoid cell populations. (**D**) Percentages of the indicated cell subsets in the thymus of mice of the indicated genotypes. (**E-G**) Complementary data for Figure 1D-E, H to include single knockout mice as controls. (**H**) Representative FACS plots of cells from wt mice indicate gating strategy to identify cell populations of interest in the peripheral blood. B cells (B220^+^), T cells (TCRβ^+^) and myeloid cells (MAC1^+^B220^-^TCRβ^-^). (**I**) Percentages of the indicated cell subsets in the peripheral blood of mice of the indicated genotypes.

**Supplementary Figure 2. Major organ analysis of young adult mice of the indicated genotypes.**

Representative H&E-stained sections of the indicated organs, as indicated, from 8- to 12-week old mice of the indicated genotypes. N represents number of mice analysed. Scale bar denotes 500 µm.

**Supplementary Figure 3 RNA sequencing of thymocytes from young adult TKO mice**

RNAseq analysis of isolated thymocytes from 8-12 week old wt (N=4), *Zmat3^-/-^* (N=4) and *Puma^-/-^p21^-/-^Zmat3^-/-^* TKO (N=3) mice. (**A**) Functional enrichment analysis of the biological processes was conducted using the Gene Ontology (GO) database. Significant GO terms are shown with an associated p-adjusted value (determined by circle colour) and GeneRatio (Number of differentially abundant proteins associated with the GO terms / number of input differentially abundant proteins). The circle size is given by the count of proteins detected that are involved in each GO term. (**B**, **C**) Volcano plot of deferentially expressed genes between cells from mice of the indicated genotypes. Genes with a Log2FC cut off 1.5 and p.adj<0.05 were considered significant (red dots). (**B**) Known TP53/TRP53 regulators and target genes are labelled. (**C**) Comparing cells from *Zmat3^-/-^* mice with cells from TKO mice shows only 9 DE genes (labelled).

**Supplementary Figure 4. Characterisation of γ-radiation induced thymic lymphomas.**

(**A**) Representative FACS plots from immunophenotyping of γ-radiation-induced thymic lymphomas arising in mice of the indicated genotypes as assessed by cell surface marker staining and flow cytometric analysis. Lymphoma cells found in the thymus were classified as either 1) CD4^-^CD8^-^ double negative / CD4^-^CD8^+^ single positive mixed (DN / CD8^+^), 2) CD4^-^CD8^+^ single positive (CD8^+^), 3) CD4^-^CD8^+^ single positive and CD4^+^CD8^+^ double positive mixed (DP / CD8^+^) or 4) CD4^+^CD8^+^double positive only (DP). (**B**) Spleen weights and (**C**) white blood cell counts (WBC) in peripheral blood from sick mice of the indicated genotypes. Mean ± SEM, Unpaired Students t-test. p value indicated compares sick *p21^-/-^Zmat3^-/-^* and sick wt γ-irradiated mice. *p21^-/-^Zmat3^-/-^* (N=12), *p21^-/-^* (N=7), *Zmat3^-/-^* (N=7) and wt (N=5).

**Supplementary Figure 5. Histological analysis of sick *Puma^-/-^p21^-/-^Zmat3^-/-^* mice.**

Representative H&E-stained sections of the indicated organs that show evidence of tumours from sick *Puma^-/-^p21^-/-^Zmat3^-/-^* mice with indicated mouse numbers. Age-matched wt mice were used for comparison (for summary of the histopathology report see Table 1). Scale bar denotes 500 µm.
