# Supplementary figures and images for: Combined absence of TRP53 target genes ZMAT3, PUMA and p21 cause a high incidence of cancer in mice
Source: Cell Death Differ. 2023 Dec 18;31(2):159–69. doi: 10.1038/s41418-023-01250-w (PMC10850490; doi:10.1038/s41418-023-01250-w)

# SUPP. FIGURE 1

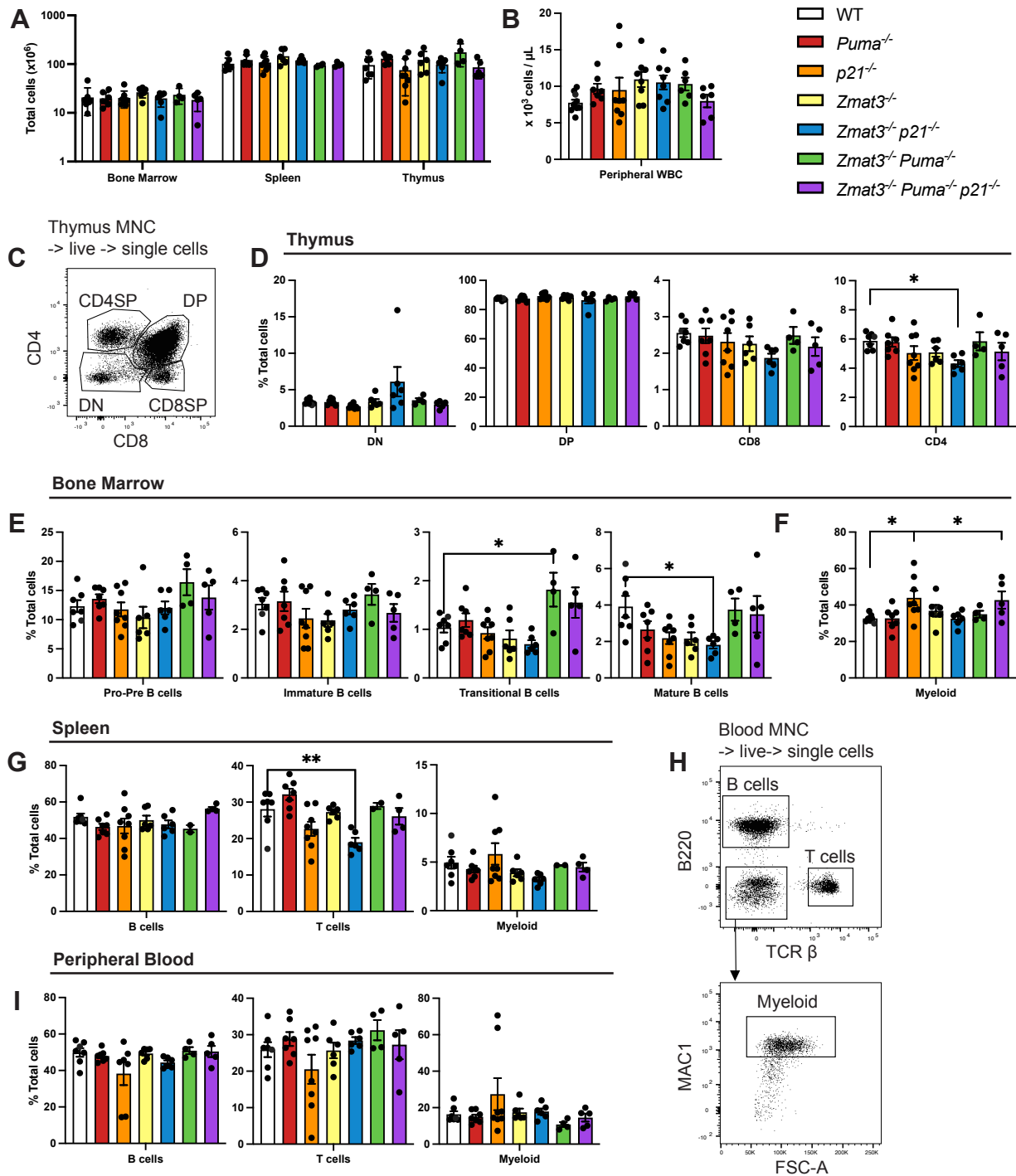

Supplement: Supplementary file 2 — Supplemental Figure 1 [file 41418_2023_1250_MOESM2_ESM.pdf]

SUPP. FIGURE 2

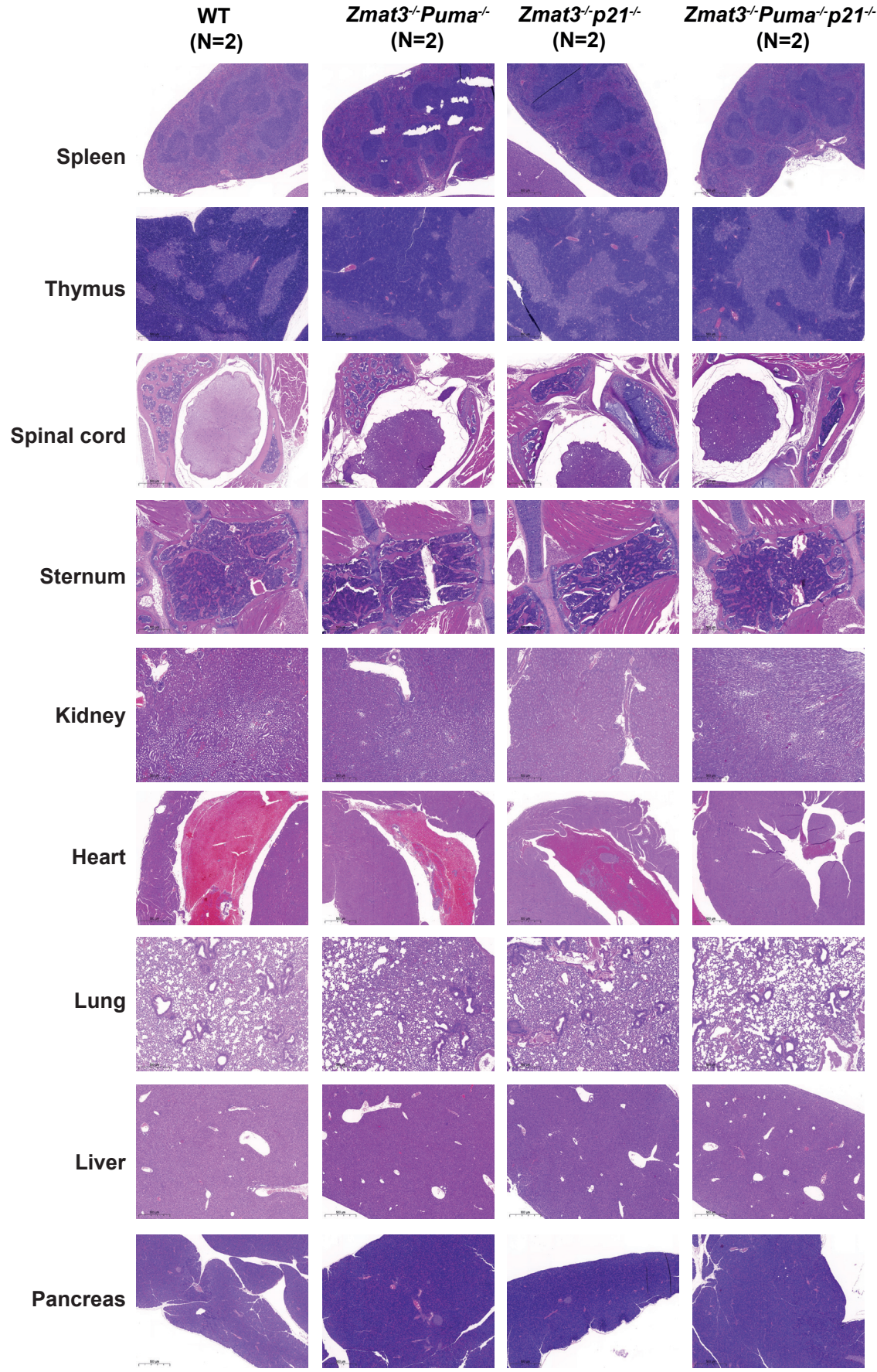

Supplement: Supplementary file 3 — Supplemental Figure 2 [file 41418_2023_1250_MOESM3_ESM.pdf]

SUPP. FIGURE 3

A

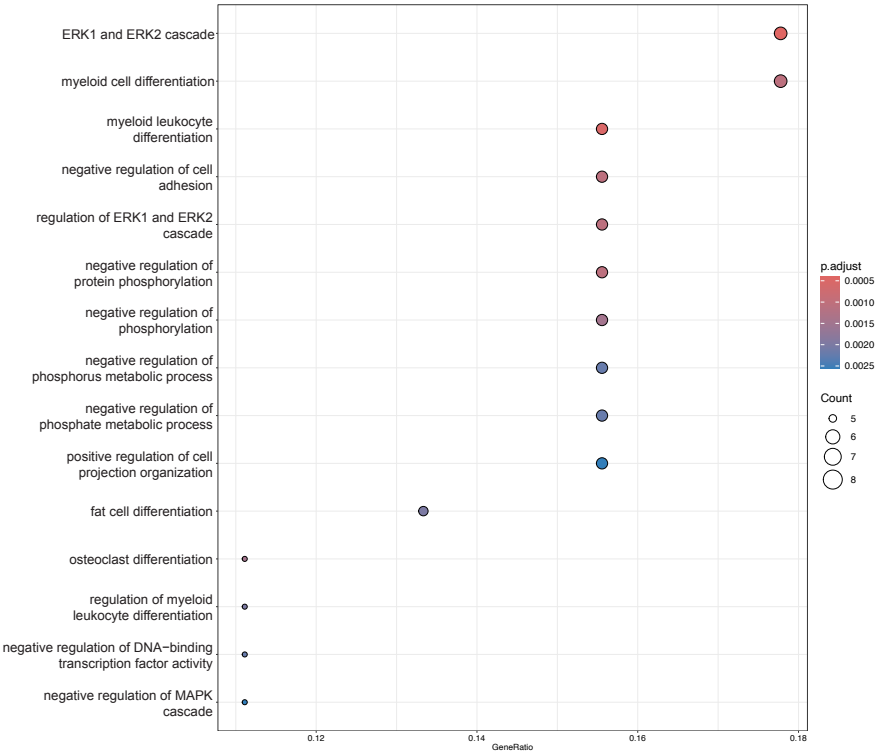

B

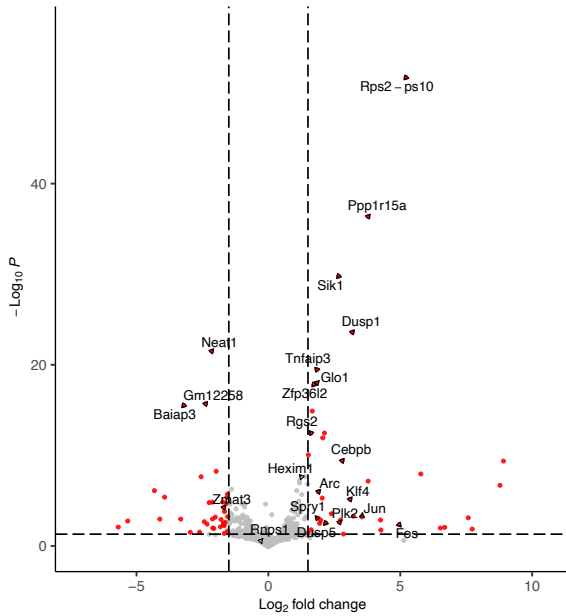

C

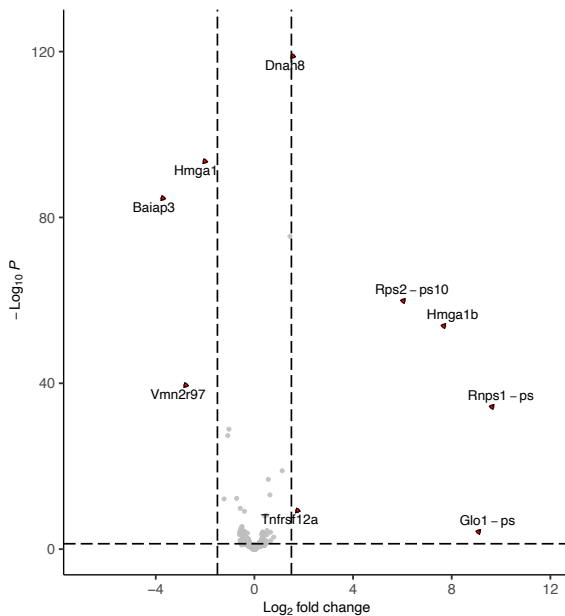

Supplement: Supplementary file 4 — Supplemental Figure 3 [file 41418_2023_1250_MOESM4_ESM.pdf]

# SUPP. FIGURE 4

**A** Thymocytes  
-> live -> single cells

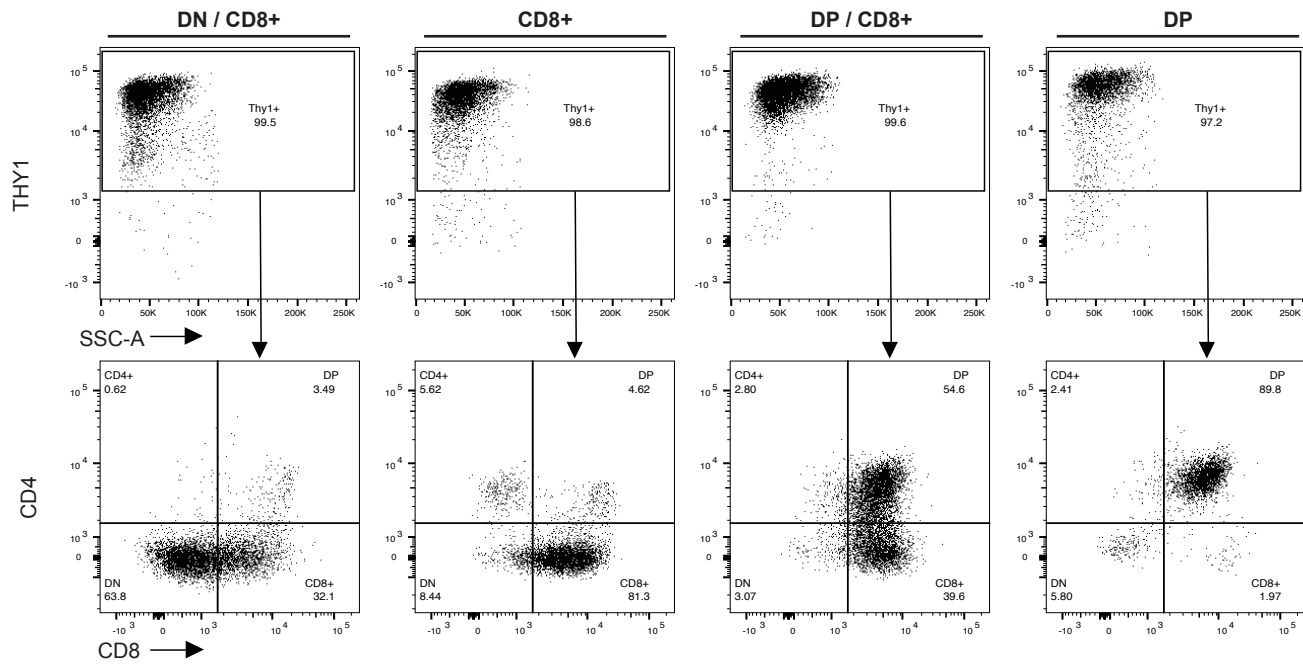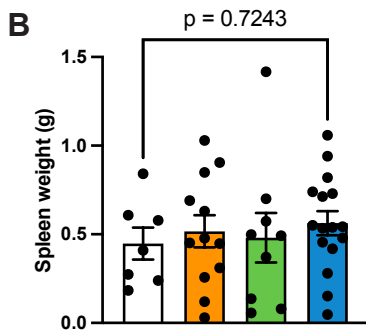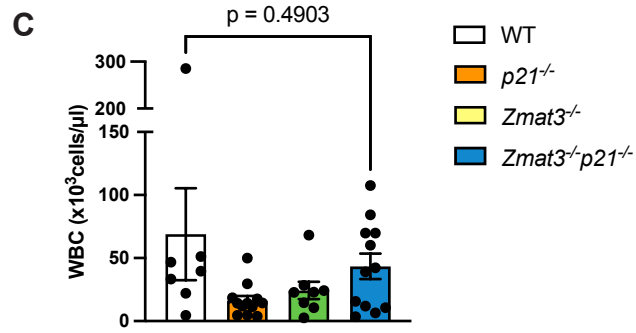

WT  
 $p21^{-/-}$   
 $Zmat3^{-/-}$   
 $Zmat3^{-/-}p21^{-/-}$

Supplement: Supplementary file 5 — Supplemental Figure 4 [file 41418_2023_1250_MOESM5_ESM.pdf]

SUPP. FIGURE 5

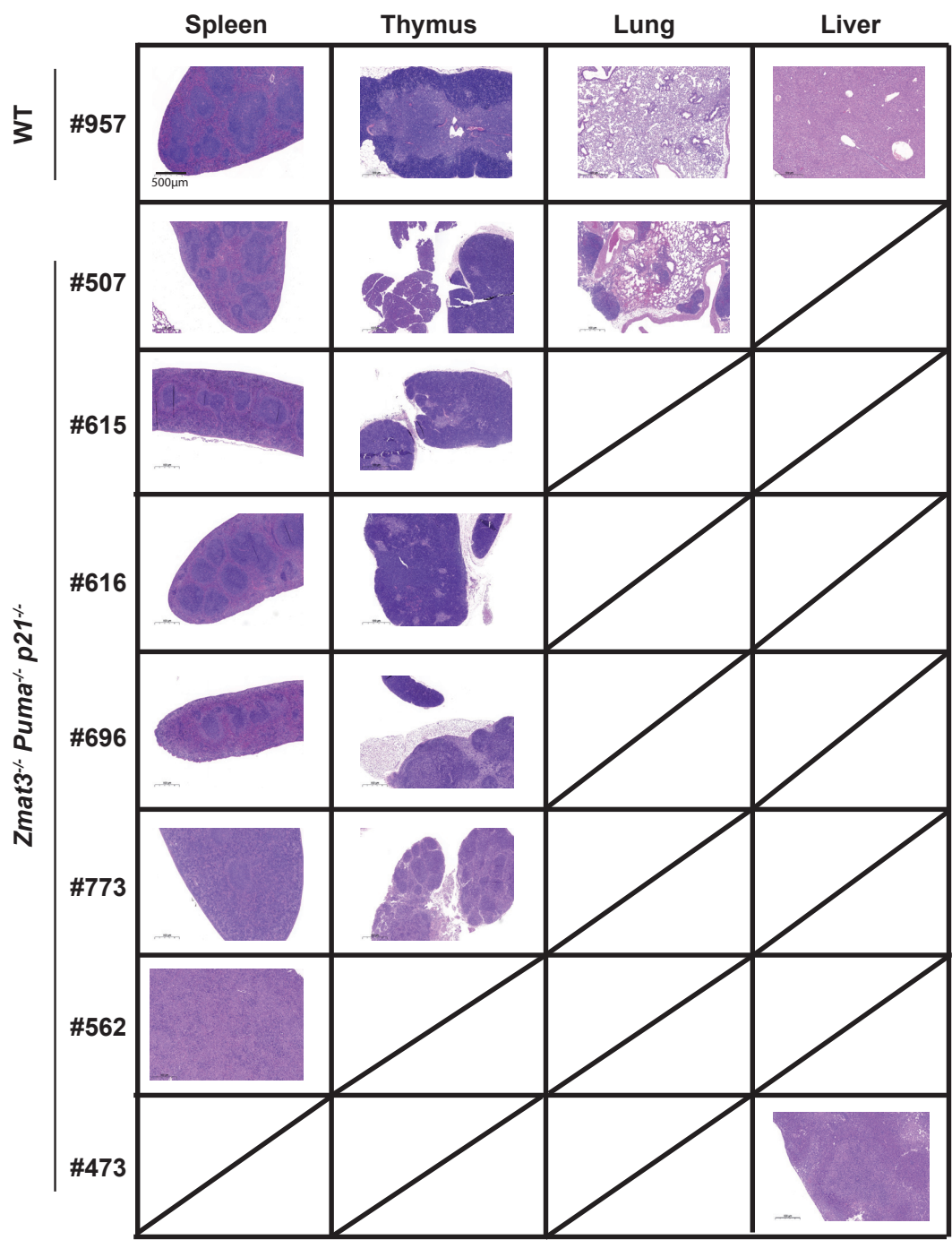

Supplement: Supplementary file 6 — Supplemental Figure 5 [file 41418_2023_1250_MOESM6_ESM.pdf]
