# Supplementary material for: Combined absence of TRP53 target genes ZMAT3, PUMA and p21 cause a high incidence of cancer in mice
Source: Cell Death Differ. 2023 Dec 18;31(2):159–69. doi: 10.1038/s41418-023-01250-w (PMC10850490; doi:10.1038/s41418-023-01250-w)
Supplement: Supplementary file 7 — Supplemental Table 1 [file 41418_2023_1250_MOESM7_ESM.docx]

**Table S1:** Mendelian ratios from heterozygous crosses

|  |  | Offspring obs.(exp.) | | |  |  |  |
| --- | --- | --- | --- | --- | --- | --- | --- |
| Breeding pair | **+/+** | | **+/-** | **-/-** | | ***X^2^*** | **p** |
| *Zmat3^+/-^* X *Zmat3^+/-^* | 63 (62.75) | | 129(125.5) | 59(62.75) | | 0.3227 | 0.8510 |
| *Puma^+/-^* X *Puma ^+/-^* | 137 (148.5) | | 317(297) | 140(148.5) | | 2.724 | 0.2562 |
| *p21^+/-^* X *p21^+/-^* | 37(29.25) | | 58(58.5) | 22(29.25) | | 3.855 | 0.1455 |

Observed (obs) and expected (exp) Mendelian inheritence of induvidual alleles within *Zmat3;puma;p21* intercrossed colony at weaning. Data include heterozygous intercrosses of each allele irrespective of other allele genotypes. Chi-square test (degrees freedom = 2) was used to determine p-value. p>0.05 = not significant.

**Table S2:** Mendelian ratios from di-hybrid crosses

|  |  |  |  |  | **Offspring obs.(exp.)** | | |  |  | |  | | |  |
| --- | --- | --- | --- | --- | --- | --- | --- | --- | --- | --- | --- | --- | --- | --- |
|  | **+/+**  **+/+** | **+/+**  **+/-** | **+/+**  **-/-** | **+/-**  **+/+** | **+/-**  **+/-** | **+/-**  **-/-** | **-/-**  **+/+** | **-/-**  **+/-** | | **-/-**  **-/-** | | ***X^2^*** | **p** | |
| **Breeding pair** |  |  |  |  |  |  |  |  |  |  |  |  |  |  |
| *Zmat3^+/-^;puma^+/-^* X *Zmat3^+/-^;puma^+/-^* | 10  (10.9) | 17  (21.9) | 14  (10.8) | 23  (21.9) | 44  (43.7) | 23  (21.9) | 14  (10.9) | 22  (21.9) | | 8  (10.9) | | 3.789 | 0.8757 | |
| *Zmat3^+/-^;p21^+/-^* X *Zmat3^+/-^;p21^+/-^* | 8  (7) | 14  (14) | 7  (7) | 23  (14) | 32  (28) | 8  (14) | 5  (7) | 10  (14) | | 5  (7) | | 11.36 | 0.1823 | |

Observed (obs) and expected (exp) Mendelian inheritence of compound *Zmat3^+/-^;puma^+/-^* and *Zmat3^+/-^p21^+/-^* intercrosses. Chi-square test (degrees freedom = 8) was used to determine p-value. p>0.05 = not significant.

**Table S3: DE genes in attached excel spreadsheet**

**Table S4. Dysregulation of p53 pathway in irradiation induced thymic lymphoma model**

| **Animal #** | **Western blot** | **Sequencing** |
| --- | --- | --- |
| **Wild type** | |  |
| 886 | N.D | +/+ |
| 889 | + | mut/- |
| 914 | - | +/+ |
| 915 | + | +/+ |
| 892 | - | +/+ |
| ***p21^-/-^*** | |  |
| 890 | + | +/+ |
| 910 | + | mut/+ |
| 911 | - | +/+ |
| 997 | N.D | +/+ |
| 998 | N.D | +/+ |
| ***Zmat3^-/-^*** | |  |
| 894 | - | +/+ |
| 896 | - | +/+ |
| 930 | + | mut/+ |
| 931 | + | +/+ |
| 932 | - | +/+ |
| 934 | - | N.D |
| ***Zmat3^-/-^ p21^-/-^*** | |  |
| 884 | N.D | +/+ |
| 885 | N.D | +/+ |
| 900 | N.D | +/+ |
| 901 | - | +/+ |
| 904 | - | +/+ |
| 937 | - | +/+ |
| 939 | N.D | mut/+ |
| 942 | - | +/+ |
| 943 | - | +/+ |
| 944 | - | +/+ |
| 945 | - | +/+ |

N.D: Not done

**Table S5:** Genotyping primers

| Allele | Primers | Fragment size (bp) |
| --- | --- | --- |
| *Zmat3* (wildtype) | 5’TGGTCCATTACTTGGTTGGAC  5’AGTGGTTCTGGAAGGCAGAG | 416 |
| *Zmat3* (knockout) | 5'TGATTTTGCAAGGGCTGTAG  5’CCACACTGAACATGGTGTGAC | 308 |
| *Puma* | 5’ACCGCGGGCTCCGAGTAGC  5’GGACTGTCGCGGGCTAGACCCTCTG  5’AGGCTGTCCCTGGGGTCATCCC | 203 (WT)  379 (knockout) |
| *p21* | 5’AAGCCTTGATTCTGATGTGGGC  5’TGACGAAGTCAAAGTTCCACC  5’GCTATCAGGACATAGCGTTGGC | 872 (WT)  700 (knockout) |
