# Supplementary material for: Combined absence of TRP53 target genes ZMAT3, PUMA and p21 cause a high incidence of cancer in mice
Source: Cell Death Differ. 2023 Dec 18;31(2):159–69. doi: 10.1038/s41418-023-01250-w (PMC10850490; doi:10.1038/s41418-023-01250-w)
Supplement: Supplementary file 9 — Original Data File [file 41418_2023_1250_MOESM9_ESM.pdf]

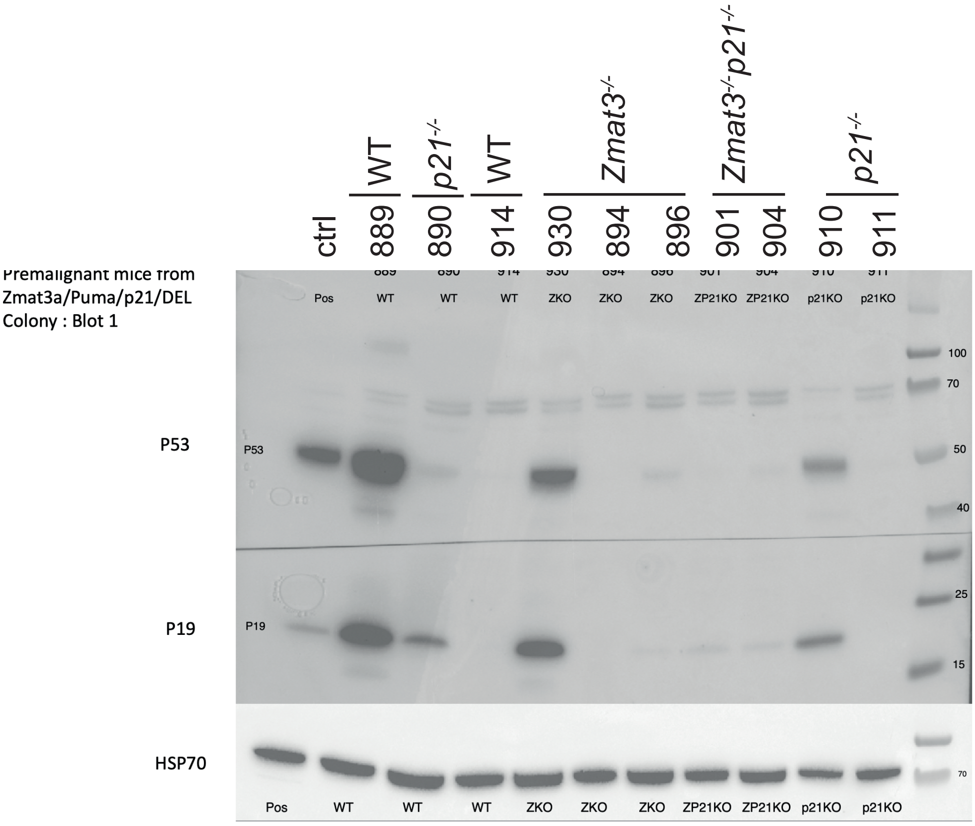
ctrl
889
890
914
930
894
896
901
904
910
911
WT
p21-/-
WT
Zmat3-/-
Zmat3-/-p21-/-
p21-/-
Premalignant mice from
Zmat3a/Puma/p21/DEL
Colony : Blot 1
Pos
WT
WT
WT
ZKO
ZKO
ZKO
ZP21KO
ZP21KO
p21KO
p21KO
P53
P19
HSP70
100
70
50
40
25
15
70
Pos
WT
WT
WT
ZKO
ZKO
ZKO
ZP21KO
ZP21KO
p21KO
p21KO

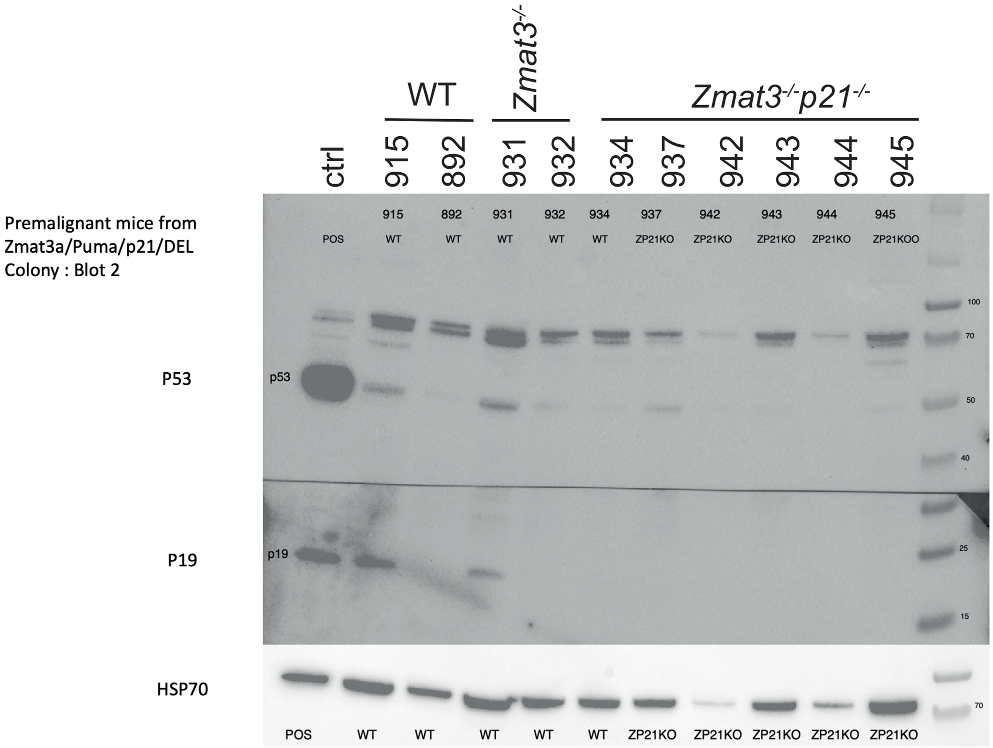
ctrl
915
892
931
932
934
937
942
943
944
945
WT
Zmat3-/-
Zmat3-/-p21-/-
Premalignant mice from
Zmat3a/Puma/p21/DEL
Colony : Blot 2
915
892
931
932
934
937
942
943
944
945
POS
WT
WT
WT
WT
WT
ZP21KO
ZP21KO
ZP21KO
ZP21KO
ZP21KOO
P53
p53
P19
p19
HSP70
100
70
50
40
25
15
70
POS
WT
WT
WT
WT
WT
ZP21KO
ZP21KO
ZP21KO
ZP21KO
ZP21KO
